# Sequential use of real-time polymerase chain reaction and enzyme-linked immunosorbent assay techniques verifies adulteration of fermented sausages with chicken meat

**DOI:** 10.5713/ab.21.0139

**Published:** 2021-06-23

**Authors:** Hakan Benli, Elif Barutcu

**Affiliations:** 1 Department of Food Engineering, Cukurova University, Adana 01330, Turkey

**Keywords:** Adulteration, Chicken Meat, Enzyme-linked Immunosorbent Assay (ELISA), Fermented Sausage, Real-time Polymerase Chain Reaction (real-time PCR)

## Abstract

**Objective:**

Detection of adulteration in processed meats is an important issue for some countries due to substitution of beef with a cheaper source of protein like poultry. In this study, the presence of chicken meat was investigated using real-time polymerase chain reaction (real-time PCR) and enzyme-linked immunosorbent assay (ELISA) techniques to verify adulteration of fermented sausage samples.

**Methods:**

A total of 60 commercial samples were collected from 20 establishments in three replicates including 10 fermented sausage manufacturers and 10 butchers to investigate the presence of chicken meat with the sequential use of real-time PCR and ELISA techniques. In addition, pH, moisture content, water activity and color values of the samples were determined.

**Results:**

Both real-time PCR and ELISA showed agreement on the presence or absence of chicken meat in 55 out of 60 fermented sausage samples and chicken meat was identified with both methods in 16 samples. Five samples produced inconsistent results for the presence of chicken meat in the first run. Nevertheless, the presence of chicken meat was verified with both methods when these samples were analyzed for the second time. In addition, the average physico-chemical values of the fermented sausage samples tested positive for chicken meat were not significantly different from some of those fermented sausage samples tested negative for the chicken meat.

**Conclusion:**

The sequential use of real-time PCR and ELISA techniques in fermented sausages could be beneficial for the government testing programs to eliminate false negatives for detection of adulteration with chicken meat. Furthermore, consumers should not rely on some of the quality cues including color to predict the adulteration of fermented sausages with chicken meat since there were no statistical differences among some of the samples tested positive and negative for chicken meat.

## INTRODUCTION

Food authenticity and adulteration are among the issues concerning consumers [1]. Authentication of food ingredients is important for human health due to allergenic and toxic substances. In addition, consumers demand to know the ingredients in their foods because of their religious beliefs or preferences [2]. Many countries require meat species must be clearly labelled for processed meat products to inform their consumers. Although meat species are mostly distinguishable for wholesale cuts, the authentication of the species requires more detailed analysis in processed meats due to changes in texture, flavor and color [3]. Nevertheless, intentional adulteration of processed meats to gain economic advantages has been practiced by some producers all around the world [4–6]. Combining an expensive meat such as beef with a cheaper source of protein such as poultry or pork has been a significant issue for countries like Korea, Japan, China, and Turkey [2]. Thus, combining beef with poultry or any other type of meat is prohibited when preparing processed meats including beef sucuk in Turkey due to the concerns of the intentional adulteration [7]. Traditional Turkish sucuk is considered a dry or semi-dry, fermented sausage produced without any heat treatment. However, heat treated sucuks are also available in the market and produced following a short period of fermentation (12 to 24 h) and a drying step [8].

Fast, accurate and reliable analysis methods have been developed to detect the intentional or unintentional adulteration related to the use of different types of meat in processed meats. Immunological, electrophoretic, serological, and genetic methods are among these methods although real-time polymerase chain reaction (real-time PCR) and enzyme-linked immunosorbent assay (ELISA) techniques are the most preferred methods due to their sensitivity, accuracy, reliability, and rapid results [3,9,10]. The PCR is a method based on the amplification of a specific sequence of DNA. The number of copies of the target sequence is increased exponentially in each cycle of the PCR to produce millions of copies after repeating the cycles numerous times. Thermal cycling is applied for melting the DNA into single strands and replicating the target sequence via enzymatic replication. In addition, real-time PCR method is a quantitative PCR method that can be achieved by measuring the fluorescence signal that increases simultaneously with the amplification. The real-time PCR method uses an optical reading system and probes consisting of synthetic oligonucleotides. When PCR amplification is performed, the probes form fluorescent radiation by binding amplified target DNA strands. They have been designed to fit the specific sequence of the target DNA to be able to produce fluorescent radiation [9–12]. ELISA is a protein-based immunological technique in which a soluble antigen or antibody is bound to a solid surface (immunosorbent), such as a 96-well plastic microtiter plate. Then a blocking buffer including a nonspecific protein like bovine serum albumin is applied to block the remaining uncoated surface on the plate. Different immunoassay reagents can be used to incubate for a specified time and at a temperature. The coated surface is then washed to separate free or unbound molecules from bound molecules. A colored end product is obtained due to the conversion of a colorless substrate to a colored product by an enzyme. The developed color can be detected visually or spectrophotometrically [12].

Real-time PCR and ELISA techniques are extremely sensitive identifying meat species in mixtures [3,9]. Although this sensitivity could be advantageous for detecting different meat species in processed products, it could also create some problems due to the cross contamination since most of the manufacturers produce beef and poultry products in the same facilities. Therefore, in Turkey, public and private laboratories have been using the ELISA method to search for poultry meat and use the real-time PCR method as a verification approach. Thus, the aim of this study was to investigate the presence of chicken meat by sequential use of real-time PCR and ELISA techniques to effectively verify the detection of adulteration in sucuk samples. In addition, pH, moisture content, water activity, and color values of the samples were investigated.

## MATERIALS AND METHODS

A total of 60 beef fermented sausage samples were collected from 20 establishments in three replicates considering different production dates. Thirty of the samples were collected from 10 establishments which had business approval certificates in accordance with the Turkish Food Codex Communiqué on Meat, Prepared Meat Mixtures, and Meat Products (Communiqué No: 2018/52) for sucuk production. In addition, a total of 30 samples produced with the traditional method were collected from 10 different butchers located in the areas known to have intensive sucuk production in Adana region. However, none of these butchers had a business approval certificate for sucuk production.

### DNA extraction and quantification

DNA extractions were performed from 100 mg of homogenized sucuk sample using the Nucleospin Blood kit (Macherey-Nagel GmbH & Co. KG, Dueren, Germany) according to the manufacturer’s instructions. Lysis was accomplished by incubating at 65°C for 30 min using a dry block thermostat (Boeco, TDB-100, Hamburg, Germany). Preheated elution buffer BE (70°C) was used to elute DNA. The purity and concentration (ng/μL) of the resulting DNA was measured using BioSpec-nano spectrophotometer (Shimadzu, Japan).

### Real-time polymerase chain reaction

The samples were treated with real-time PCR Kit for Chicken Species Detection (SNP Biotechnology, Ankara, Turkey) and qualitatively analyzed using a Real-time PCR (Applied Biosystems 7500 Fast, Thermoscientific, Foster City, CA, USA) according to the kit’s protocol. The PCR amplification cycles included an initial denaturation at 95°C for 10 min, followed by 30 cycles of 95°C for 15 s and 60°C for 1 min. Positive and negative controls were included for each PCR run. After the end of the PCR procedure, the amplifications obtained with the target gene dye FAM (dye absorbing 494 and 515 nm wavelengths) and the internal control dye HEX (dye absorbing 535 and 555 nm wavelengths) were evaluated. The results were presented as the presence or absence of chicken meat in the fermented sausage samples according to Ct (cycle threshold) values.

### Enzyme-linked immunosorbent assay

Fermented sausage samples were extracted, and the poultry meat was determined with the species-specific type of sandwich ELISA test kit (ELISA-TEK Cooked Meat Poultry Species Kit, ELISA Technologies, Inc., Gainesville, FL, USA). Approximately 20 g of homogenized samples were weighed for the extraction. Then 40 mL of 0.9% sodium chloride solution was added and samples were subjected to heat treatment in a microwave oven (Vestel, MD,700 W, Istanbul, Turkey) for 2 min. Approximately 1 mL was taken into 1.5 mL capped tubes from the upper phase of the samples after cooling down to the room temperature. The tubes were then centrifuged at 13,000 rpm for 2 min (Eppendorf, 5424, Enfield, CT, USA). The supernatant was transferred into 1.5 mL tubes to obtain the extract. The extracts then treated according to manufacturer’s instruction before the absorbance values were taken at 414 and 492 nm wavelengths using an ELISA reader (Thermo, Multiscan Spectrum, Waltham, MA, USA) and the results were determined as the presence or absence of chicken meat in the fermented sausage samples.

### pH, moisture content, and water activity

pH values in the slurries were measured using a calibrated pH meter (S220, Mettler-Toledo, LLC, Columbus, OH, USA). The samples were dried in drying oven (Memmert, Universal Oven Tech., Schwabach, Germany) at 100°C for 16 h to determine the moisture contents [12,13]. A calibrated instrument of LabMASTER-aw system (Novasina, Lachen, Switzerland) was used to determine water activity (a_w_) values of the samples.

### Color analysis

A Konica Minolta Colorimeter (CR-400, Minolta C., Ramsey, NJ, USA) was used to take color space values of L* (lightness), a* (redness), and b* (yellowness) from the outer and inner surfaces of samples. The colorimeter equipped with an 8 mm aperture size was calibrated using a standard white tile (Y = 93.7, x = 0.3157, y = 0.3323) with the setting of illuminant D-65 and 2° observer. Three random measurements were taken from different locations of outer and inner surfaces [14].

### Statistical analyses

Data were statistically analyzed using SPSS software version 20 (IBM SPSS Statistics, Armonk, NY, USA). Significant differences were determined using analysis of variance (One-way) procedures and Tukey multiple comparison test.

## RESULTS AND DISCUSSION

### Real-time polymerase chain reaction and enzyme-linked immunosorbent assay

Both real-time PCR and ELISA results agreed on the presence or absence of chicken meat in 55 out of 60 fermented sausage samples (Table 1). Among the 55 samples, the presence of chicken meat was detected in 16 samples with both methods. Thus, both real-time PCR and ELISA results indicated that the second and third samples of Manufacturers 3 and 8 and all three samples of Butchers 6, 7, 8, and 9 were adulterated with chicken meat.

Conversely, five samples produced inconsistent results for the presence of chicken meat with both methods. ELISA results indicated the presence of chicken meat in the first samples of Manufacturer 3 and 8 and all three samples of Manufacturer 5. However, the presence of chicken meat was not detected in these samples by real-time PCR in the first run. Nevertheless, all these 5 controversial samples were analyzed for the second time using real-time PCR and ELISA methods to verify the results (Table 2). The results indicated the presence of chicken meat in all five samples and verified the adulteration of all samples collected from Manufacturers 3, 5, and 8. These results suggested that sequential use of real-time PCR and ELISA techniques could be beneficial to verify adulteration of fermented sausages with chicken meat to eliminate false negatives. Similarly, Perestam et al [3] reported that using both real-time PCR and ELISA techniques together could be a beneficial approach for identification of species in meat products that contain additional ingredients [3]. Furthermore, the inconsistent results obtained in the first run could be due to a number of reasons including the presence of some inhibitory ingredients, amount of the sample used for the testing and difficulties to determine species in high fat processed meats. The extreme sensitivity of the real time PCR technique is mentioned in the literature [3,15,16]. However, it was also reported that the mixing distributes the ingredients through the sausage in an unpredictable manner [17]. Thus, addition or cross contamination of small amounts of chicken meat might not be distributed thoroughly in sausages. Consequently, the lower amount of sample used (100 mg) in the real-time PCR technique could have reduced the adequate representation of the samples in the current study. Conversely, the amount of sample (20 g) used in the ELISA method was thought to affect the results since it might have represented the sample more adequately than the real-time PCR method.

Moreover, the samples collected from 4 out of 10 butchers (Butchers 6, 7, 8, and 9) were positive for the presence of chicken meat when analyzed with both real-time PCR and ELISA methods in the first run. This might be an indication of intentional adulteration of the products with chicken meat. Chicken meat is a cheaper alternative to beef for manufacturing sucuk in Turkey. In addition, none of the butchers had business approval certificates to produce sucuk, thus their products might not be tested regularly by the government agency for the presence of chicken meat. Conversely, real-time PCR was only able to positively identify chicken meat in 4 out of 9 samples collected from 3 manufacturers (Manufacturer 3, 5, and 8) in the first run. However, all three samples collected from those 3 manufacturers were positive for the presence of chicken meat with ELISA. All these manufacturers had business approval certificates to produce sucuk and their products regularly tested for the presence of chicken meat according to government testing program. Thus, failure of real-time PCR to detect chicken meat in the first run might be an indication of an unintentional adulteration of the products with chicken meat. The production of sucuk using only chicken meat (chicken meat sucuk) is not prohibited according to Turkish Food Codex Communiqué on Meat, Prepared Meat Mixtures, and Meat Products (Communiqué No: 2018/52). Therefore, cross contamination could be the reason during the production of different types of fermented sausages using the same equipment or processing line.

### Physico-chemical analyses

Table 3 shows the mean pH, moisture, and water activity (a_w_) values of fermented sausage samples. Butcher 1 (4.64) and Manufacturer 9 (6.50) had the lowest and highest pH values, respectively (p<0.05). pH values were highly variable regardless of the producer (manufacturer or butcher) and the type of sucuk (heat treated or traditional). In a study, Gencelep et al [18] reported that pH values were between 4.53 and 6.29 for 30 sucuk samples purchased from different cities. Similarly, Siriken et al [19] collected 100 sucuk samples from Afyon province and reported that pH values were between 4.84 and 6.50. Benli [8] also indicated a higher variation among the pH values of 36 sucuk samples purchased from Adana province ranging from 4.69 to 6.56. High pH values observed in sucuk samples might be related to use of DFD (dark, firm, and dry) meat which had an ultimate pH in the range of 6.5 to 6.8 and/or poor manufacturing techniques [20]. Since lactic acid formation is essential for the fermented sausage products [21], the previous studies and the current study indicated a lack of standardized methods for sucuk production in Turkey.

Manufacturers 7 and 9 had the highest average moisture contents of 46.52% and 47.86%, respectively while butcher 2 had the lowest moisture content (32.72%) (p<0.05). All the fermented sausage samples produced by the manufacturers with a business approval certificate (except manufacturer 2) were heat treated sucuks and the average moisture contents of the samples were less than the specified maximum value (50%) in Turkish Food Codex, Meat and Meat Products Communiqué (Communiqué No: 2012/74). However, the average moisture contents of the fermented sausage samples produced with a traditional method by seven butchers and manufacturer 2 were above the specified maximum value (40%) in the communiqué, although some values were close to the limit.

Butcher 2 and manufacturer 9 had the lowest (0.8580) and highest (0.9100) mean aw values, respectively (p<0.05). Gencelep et al [18] were reported that aw values of the sucuk samples were between 0.761 and 0.960. The results reported in the current study and the previous studies were also indicated a higher variation and a lack of standardized production methods for the sucuk manufacturing among the producers.

Consumers are influenced by the expected product quality of food products before making a buying decision. The quality cues are observed by consumers during shopping for the prediction of the quality performance of that product while consuming. As an intrinsic cue, the color is among the most important quality cues for consumer acceptability of the meat products [22]. The mean outer and inner surface L* values of fermented sausage samples ranged between 37.20 to 47.76 and 42.81 to 52.44, respectively (Tables 4, 5). The mean outer and inner surface a* values of samples ranged between 5.31 to 18.55 and 14.25 to 26.36, respectively. The mean outer and inner surface b* values of samples ranged between 14.31 to 28.31 and 24.34 to 37.18, respectively. Although the color is among the most important quality cues for consumer acceptability of the meat products, outer and inner surface L*, a*, b* values of fermented sausage samples tested positive for chicken meat were not significantly different from some of those samples tested negative for the chicken meat. Thus, the consumers might not be able to detect any adulteration of sucuks with chicken meat by visually inspecting the products during the purchase.

## CONCLUSION

Fermented sausage samples collected from local manufacturers and butchers were tested using real-time PCR and ELISA techniques to verify detection of chicken meat. In the first run, both real-time PCR and ELISA methods identified the presence of chicken meat in all samples of 4 out of 10 butchers (Butchers 6, 7, 8, and 9) indicating an intentional adulteration. Although five samples produced inconsistent results in the first run, all samples were tested positive for the presence of the chicken meat in the second run. All of five controversial samples were collected from the fermented sausage manufacturers with business approval certificates. Since these manufacturers’ products were tested for the presence of chicken meat regularly by the government agency, the controversial results might be an indication of unintentional adulteration of the products with chicken meat due the cross contamination. However, these manufacturers would be still considered as adulterating their products intentionally since there is a zero tolerance policy for the presence of the chicken meat in beef sucuks. In addition, physico-chemical analyses indicated higher variabilities regardless of the producer (manufacturer or butcher) and the type of sucuk (heat treated or traditional) among the sucuk samples. As a conclusion, a sequential use of real-time PCR and ELISA techniques in fermented sausages could be beneficial for government testing programs to eliminate false negatives for detection of chicken meat adulteration.

## Figures and Tables

**Table 1:** Real-time PCR and ELISA results of a total of 60 sucuk samples

Sucuk	Sucuk type	Real-time PCR results			ELISA results		
	
		Sample 1	Sample 2	Sample 3	Sample 1	Sample 2	Sample 3
Manufacturer 1	Heat treated	−	−	−	−	−	−
Manufacturer 2	Traditional	−	−	−	−	−	−
Manufacturer 3	Heat treated	−	+	+	+	+	+
Manufacturer 4	Heat treated	−	−	−	−	−	−
Manufacturer 5	Heat treated	−	−	−	+	+	+
Manufacturer 6	Heat treated	−	−	−	−	−	−
Manufacturer 7	Heat treated	−	−	−	−	−	−
Manufacturer 8	Heat treated	−	+	+	+	+	+
Manufacturer 9	Heat treated	−	−	−	−	−	−
Manufacturer 10	Heat treated	−	−	−	−	−	−
Butcher 1	Traditional	−	−	−	−	−	−
Butcher 2	Traditional	−	−	−	−	−	−
Butcher 3	Traditional	−	−	−	−	−	−
Butcher 4	Traditional	−	−	−	−	−	−
Butcher 5	Traditional	−	−	−	−	−	−
Butcher 6	Traditional	+	+	+	+	+	+
Butcher 7	Traditional	+	+	+	+	+	+
Butcher 8	Traditional	+	+	+	+	+	+
Butcher 9	Traditional	+	+	+	+	+	+
Butcher 10	Traditional	−	−	−	−	−	−

PCR, polymerase chain reaction; ELISA, enzyme-linked immunosorbent assay.

**Table 2:** Real-time PCR and ELISA results of 5 controversial sucuk samples analyzed for the second time

Sucuk	Sucuk type	Real-time PCR results			ELISA results		
	
		Sample 1	Sample 2	Sample 3	Sample 1	Sample 2	Sample 3
Manufacturer 3	Heat treated	+	NA^1)^	NA	+	NA	NA
Manufacturer 5	Heat treated	+	+	+	+	+	+
Manufacturer 8	Heat treated	+	NA	NA	+	NA	NA

PCR, polymerase chain reaction; ELISA, enzyme-linked immunosorbent assay.

1) Not available.

**Table 3:** Mean pH, moisture, and water activity values of a total of 60 sucuk samples

Sucuk	Sucuk type	pH±SD	Moisture (%)±SD	a_w_±SD
Manufacturer 1	Heat treated	4.84±0.06^ef^	45.05±0.47^abcd^	0.8820±0.0044^cd^
Manufacturer 2	Traditional	4.92±0.10^ef^	41.76±6.20^cdefg^	0.8780±0.0111^de^
Manufacturer 3	Heat treated	5.76±0.47^bc^	41.74±1.18^cdefg^	0.8833±0.0067^bcd^
Manufacturer 4	Heat treated	5.11±0.16^def^	44.72±1.57^abcd^	0.8860±0.0046^bcd^
Manufacturer 5	Heat treated	4.86±0.05^ef^	36.54±2.63^h^	0.8880±0.0053^bcd^
Manufacturer 6	Heat treated	5.07±0.15^def^	44.15±1.60^abcde^	0.9020±0.0046^abc^
Manufacturer 7	Heat treated	5.20±0.33^cdef^	46.52±0.65^a^	0.9047±0.0055^ab^
Manufacturer 8	Heat treated	4.66±0.03^f^	45.94±1.56^ab^	0.8997±0.0045^abc^
Manufacturer 9	Heat treated	6.50±0.43^a^	47.86±0.61^a^	0.9100±0.0053^a^
Manufacturer 10	Heat treated	5.01±0.02^ef^	42.58±0.62^bcdef^	0.8897±0.0032^abcd^
Butcher 1	Traditional	4.64±0.01^f^	38.07±1.87^gh^	0.8890±0.0036^abcd^
Butcher 2	Traditional	5.40±0.12^cde^	32.72±0.46^i^	0.8580±0.0200^e^
Butcher 3	Traditional	5.02±0.01^ef^	39.32±0.84^fgh^	0.8877±0.0015^bcd^
Butcher 4	Traditional	4.72±0.18^f^	44.72±1.34^abcd^	0.8890±0.0046^abcd^
Butcher 5	Traditional	4.70±0.07^f^	41.57±1.01^defg^	0.8963±0.0035^abcd^
Butcher 6	Traditional	4.68±0.03^f^	45.40±0.19^abc^	0.8923±0.0038^abcd^
Butcher 7	Traditional	4.86±0.02^ef^	42.74±0.66^bcdef^	0.8943±0.0070^abcd^
Butcher 8	Traditional	5.02±0.04^ef^	45.92±0.55^ab^	0.9043±0.0038^ab^
Butcher 9	Traditional	5.63±0.27^bcd^	40.84±0.36^^efg^^	0.8920±0.0070^abcd^
Butcher 10	Traditional	6.20±0.05^ab^	42.43±0.28^bcdef^	0.8967±0.0059^abcd^

SD, standard deviation.

a–i Means with different superscript letters are significantly different in the same column (p<0.05).

**Table 4:** Mean outer surface L*, a*, b* values of a total of 60 sucuk samples

Sucuk	Sucuk type	L*±SD	a*±SD	b*±SD
Manufacturer 1	Heat treated	43.67±1.38^bcd^	14.66±0.82^cd^	25.14±3.03^abc^
Manufacturer 2	Traditional	41.02±3.28^cdefg^	13.97±3.89^d^	21.01±4.45^de^
Manufacturer 3	Heat treated	44.68±5.37^abc^	17.32±2.51^ab^	21.57±4.43^cde^
Manufacturer 4	Heat treated	41.18±1.33^bcdef^	18.55±1.35^a^	22.54±1.37^bcd^
Manufacturer 5	Heat treated	44.44±1.76^abc^	17.14±0.96^ab^	26.49±1.71^ab^
Manufacturer 6	Heat treated	47.76±2.84^a^	15.64±0.48^bcd^	27.80±1.44^a^
Manufacturer 7	Heat treated	44.97±1.18^ab^	15.09±1.04^bcd^	28.31±2.50^a^
Manufacturer 8	Heat treated	40.53±1.50^defg^	14.41±0.79^cd^	18.99±1.83^de^
Manufacturer 9	Heat treated	39.68±2.70^efg^	16.34±1.89^abc^	19.55±3.00^de^
Manufacturer 10	Heat treated	37.75±1.20^fg^	13.92±0.89^d^	18.56±1.10^e^
Butcher 1	Traditional	38.61±1.68^efg^	14.38±1.12^cd^	21.27±2.31^cde^
Butcher 2	Traditional	37.20±1.88^g^	14.42±0.91^cd^	21.53±2.21^cde^
Butcher 3	Traditional	39.21±1.94^efg^	13.91±0.55^d^	21.33±1.73^cde^
Butcher 4	Traditional	39.42±2.74^efg^	13.86±1.09^d^	21.23±2.10^cde^
Butcher 5	Traditional	41.10±1.70^bcdef^	11.13±0.62^e^	22.88±1.59^bcd^
Butcher 6	Traditional	42.48±2.16^bcde^	14.47±0.87^cd^	20.25±1.34^de^
Butcher 7	Traditional	41.27±1.84^bcdef^	5.31±0.56^f^	14.31±1.24^f^
Butcher 8	Traditional	41.54±1.51^bcdef^	13.87±0.83^d^	20.08±1.63^de^
Butcher 9	Traditional	39.84±2.16^defg^	15.78±0.60^bcd^	21.78±1.60^cde^
Butcher 10	Traditional	44.91±1.65^abc^	16.73±2.96^abc^	26.38±2.07^ab^

SD, standard deviation.

a–g Means with different superscript letters are significantly different in the same column (p<0.05).

**Table 5:** Mean inner surface L*, a*, b* values of a total of 60 sucuk samples

Sucuk	Sucuk type	L*±SD	a*±SD	b*±SD
Manufacturer 1	Heat treated	49.62±2.00^abcd^	20.93±1.00^cd^	35.60±2.69^ab^
Manufacturer 2	Traditional	50.53±3.06^abc^	18.59±1.83^cdef^	29.66±6.75^cdef^
Manufacturer 3	Heat treated	46.68±4.65^cdef^	21.32±1.83^bc^	26.23±2.92^ef^
Manufacturer 4	Heat treated	43.78±3.59^ef^	26.36±1.94^a^	32.76±2.03^abc^
Manufacturer 5	Heat treated	52.44±1.81^a^	19.97±1.70^cde^	33.76±1.93^def^
Manufacturer 6	Heat treated	51.62±2.14^abc^	18.53±1.44^cdef^	28.99±3.64^cdef^
Manufacturer 7	Heat treated	51.73±2.48^ab^	17.39±1.67^efg^	37.18±1.57^a^
Manufacturer 8	Heat treated	48.55±1.46^abcde^	24.12±1.36^ab^	29.80±4.20^cdef^
Manufacturer 9	Heat treated	43.90±3.56^ef^	18.83±2.10^cdef^	24.34±1.83^f^
Manufacturer 10	Heat treated	51.24±0.94^abc^	17.47±2.01^ef^	30.87±4.09^bcde^
Butcher 1	Traditional	50.58±2.30^abc^	20.02±1.42^cde^	29.63±3.69^cdef^
Butcher 2	Traditional	42.81±3.33^f^	18.49±1.67^cdef^	26.30±1.72^ef^
Butcher 3	Traditional	47.49±2.74^abcdef^	19.52±1.30^cdef^	32.97±2.48^abc^
Butcher 4	Traditional	47.34±2.94^bcdef^	18.00±1.17^def^	29.11±2.79^cdef^
Butcher 5	Traditional	46.63±2.64^cdef^	14.25±1.93^g^	28.92±2.80^cdef^
Butcher 6	Traditional	49.40±5.50^abcd^	19.00±2.25^cdef^	27.19±5.24^def^
Butcher 7	Traditional	49.33±1.49^abcd^	16.46±3.61^fg^	36.26±2.29^ab^
Butcher 8	Traditional	46.88±2.85^bcdef^	20.65±2.09^cd^	31.88±2.25^abcd^
Butcher 9	Traditional	45.12±2.36^def^	20.54±1.73^cde^	28.57±1.74^cdef^
Butcher 10	Traditional	48.02±3.38^abcde^	18.19±1.44^cdef^	28.49±2.46^cdef^

SD, standard deviation.

a–g Means with different superscript letters are significantly different in the same column (p<0.05).
